# Annual Review of Statistics and Its Application: Infectious Disease Modeling

**DOI:** 10.1146/annurev-statistics-112723-034351

**Published:** 2025-03

**Authors:** Jing Huang, Jeffrey S. Morris

**Affiliations:** Department of Biostatistics, Epidemiology and Informatics, University of Pennsylvania Perelman School of Medicine, Philadelphia, Pennsylvania, USA

**Keywords:** compartmental models, time-since-infection models, agent-based models, stochastic modeling, spatial-temporal correlation, data integration

## Abstract

Infectious diseases pose a persistent challenge to public health worldwide. Recent global health crises, such as the COVID-19 pandemic and Ebola outbreaks, have underscored the vital role of infectious disease modeling in guiding public health policy and response. Infectious disease modeling is a critical tool for society, informing risk mitigation measures, prompting timely interventions, and aiding preparedness for healthcare delivery systems. This article synthesizes the current landscape of infectious disease modeling, emphasizing the integration of statistical methods in understanding and predicting the spread of infectious diseases. We begin by examining the historical context and the foundational models that have shaped the field, such as the SIR (susceptible, infectious, recovered) and SEIR (susceptible, exposed, infectious, recovered) models. Subsequently, we delve into the methodological innovations that have arisen, including stochastic modeling, network-based approaches, and the use of big data analytics. We also explore the integration of machine learning techniques in enhancing model accuracy and responsiveness. The review identifies the challenges of parameter estimation, model validation, and the incorporation of real-time data streams. Moreover, we discuss the ethical implications of modeling, such as privacy concerns and the communication of risk. The article concludes by discussing future directions for research, highlighting the need for data integration and interdisciplinary collaboration for advancing infectious disease modeling.

## 1. HISTORY OF INFECTIOUS DISEASE MODELING

The battle against infectious diseases is as old as humanity, marked by devastating outbreaks that have shaped civilizations and driven scientific advancements. The origins of infectious disease modeling are closely linked to historical pandemics such as the Black Death, smallpox, influenza, and COVID-19, underscoring the importance of understanding disease dynamics.

Mathematics has played a crucial role since the beginning. In the eighteenth century, Daniel Bernoulli (1766) was among the first to apply mathematical methods to disease modeling, specifically targeting smallpox. By using differential calculus, Bernoulli devised a model to evaluate the efficacy of variolation, a precursor to vaccination, showing how it could increase life expectancy by reducing smallpox mortality rates. This provided a logical and mathematical foundation for public health decisions regarding smallpox prevention. Over the decades, numerous mathematicians and scientists further propelled the field. In the late nineteenth century, Nobel Prize winner Ronald Ross made significant strides in understanding malaria transmission by identifying *Anopheles* mosquitoes as vectors (Ross 1897). Using differential equations, Ross mapped out the transmission cycle of malaria, introducing key concepts that led to the development of compartmental models, such as the Ross–Macdonald model (Ross 1911, Macdonald 1957). These models categorize populations into states—susceptible, infectious, and recovered—and serve as fundamental frameworks for modern epidemiological forecasting and public health planning.

The advancement of compartmental models was a landmark achievement in infectious disease modeling (Kermack & McKendrick 1927). By segmenting populations into specific states, these models became instrumental in decoding and forecasting disease transmission. Over the twentieth century, refinements included age stratification, spatial distribution, and stochastic processes, significantly enhancing their predictive accuracy and relevance to diverse epidemiological settings (Anderson & May 1985, 1991; Bartlett 1956). The twenty-first century brought computational innovations, marking a new chapter in infectious disease modeling. Simulation-based methodologies, notably agent-based models (ABMs), emerged, offering detailed perspectives on disease spread through the simulation of individual behaviors and interactions within populations (Epstein & Axtell 1996, Eubank et al. 2004). These models illuminated how social dynamics influence epidemic patterns, while time-series analysis refined our capacity for monitoring and predicting disease trends.

In recent years, incorporating real-time data, machine learning (ML), artificial intelligence (AI), and network theory into infectious disease modeling has transformed our ability to analyze complex datasets, pinpoint transmission pathways, and forecast future outbreaks with improved precision (Polgreen et al. 2008, Salathe et al. 2012, Pei et al. 2018, Peiffer-Smadja et al. 2020, Wong et al. 2023). This fusion of traditional epidemiological methods and cutting-edge computational tools has provided public health officials with powerful strategies for informed decision-making and timely intervention in disease control.

## 2. CORE MODELS AND LIMITATIONS

### 2.1. Compartmental Models

Compartmental models are a class of mathematical models that are most commonly used and crucial for understanding the dynamics of infectious disease transmission. They are termed “compartmental” because they divide the population into compartments reflecting the stages of the disease, e.g., susceptible, exposed, infectious, and recovered. Figure 1 illustrates the dynamics of two basic compartmental models.

The SIR (susceptible, infectious, recovered) model is one of the basic and simplest compartmental models (Harko et al. 2014). It is given by a set of ordinary differential equations that describe the rate of movement of individuals between compartments:

1. $$\frac{dS(t)}{dt}=-\beta \frac{S(t)I(t)}{N},$$

2. $$\frac{dI(t)}{dt}=\beta \frac{S(t)I(t)}{N}-\gamma I(t),$$

3. $$\frac{dR(t)}{dt}=\gamma I(t),$$

where S(t), I(t), and R(t) are the number of susceptible, infectious, and recovered individuals at time t, respectively; β represents the effective contact rate that leads to new infections; and γ is the recovery rate. The total population size is denoted by N and is assumed to be constant, i.e., N=S(t)+I(t)+R(t). The dynamics of the infectious group depend on both β and γ, leading to the definition of the basic reproduction number as $R_{0}=\beta ∕\gamma$.

More comprehensive compartmental models based on the SIR model can be built similarly by adding additional compartments. For example, in the SEIR (susceptible, exposed, infectious, recovered) model, the exposed (E) compartment is added to account for the incubation period of the disease, during which individuals have been infected but are not yet infectious:

$$\frac{dS(t)}{dt}=-\beta \frac{S(t)I(t)}{N},\;\frac{dE(t)}{dt}=\beta \frac{S(t)I(t)}{N}-\sigma E(t),\;\frac{dI(t)}{dt}=\sigma E(t)-\gamma I(t),\;\frac{dR(t)}{dt}=\gamma I(t),$$

with σ representing the rate at which exposed individuals become infectious (Li & Muldowney 1995). During the recent COVID-19 pandemic, more complex compartmental models emerged, incorporating compartments to better describe the transmission flows, including asymptomatic and symptomatic individuals, as well as quarantined, vaccinated, and hospitalized infections (Hao et al. 2020, Dashtbali & Mirzaie 2021).

Compared with complex compartmental models, the basic SIR model is easier to interpret and enables mathematical analysis and insights that might be difficult to obtain from complex models. For example, based on Equations 1-3, it can be shown that

$$S(t)=S(0)e^{-R_{0}\left(R(t)-R(0)\right)∕N},$$

where S(0) and R(0) represent the initial numbers of susceptible and recovered subjects, respectively. The equation implies that the number of susceptible individuals decreases over time as more individuals become infected and then recover, moving from the susceptible category to the recovered category. This decrease is dependent on the basic reproduction number $R_{0}$ and the fraction of the population that has recovered, adjusted for the total population size N. The term inside the exponential function, $-R_{0}(R(t)-R(0))∕N$, reflects the rate at which the susceptible population decreases, highlighting the impact of the disease’s spread, as captured by $R_{0}$, and the progression of the epidemic over time, as indicated by the increase in R(t) relative to R(0).

We define $s_{0}=S(0)∕N$ as the initial proportion of susceptible individuals, and $s_{\infty }=S(\infty )∕N$ and $r_{\infty }=R(\infty )∕N$ as the proportion of susceptible and recovered individuals, respectively, which describe the final state of a SIR model of an epidemic. As t approaches infinity, it can be shown that $s_{\infty }=1-r_{\infty }=s_{0}\text{exp}\{-R_{0}(r_{\infty }-r_{0})\}$. This equation can be solved using the Lambert W function, resulting in

$$s_{\infty }=1-r_{\infty }=-\frac{1}{R_{0}}W(-s_{0}R_{0}e^{-R_{0}(1-r_{0})}).$$

Such results demonstrate that under the basic assumptions of the SIR model, the end of an epidemic does not necessarily mean all individuals have been infected and recovered; rather, a proportion of the population remains susceptible provided that $s_{0}$ differs from zero. Essentially, the driving force leading to the end of an epidemic is a decline in the number of infectious individuals rather than a complete lack of susceptible individuals. On the other hand, this result also demonstrates that the role of both the basic reproduction number and the initial susceptibility are extremely important. Reorganizing the equation that describes the change in infectious individuals as $\frac{dI(t)}{dt}=\left(R_{0}\frac{S(t)}{N}-1\right)\gamma I(t)$, we find that an outbreak or a rising number of infectious individuals, $\frac{dI}{dt}(0)>0$, occurs if $R_{0}S(0)>N$. In contrast, if $R_{0}S(0)<N$, then $\frac{dI}{dt}(0)<0$, indicating that, despite the initial size of the susceptible population, a widespread epidemic outbreak is unlikely.

Moreover, Harko et al. (2014) derived an analytical solution to the SIR model, which is a set of exact, mathematical expressions that describe the behavior of the susceptible, infectious, and recovered populations over time within the framework of the SIR model without relying on numerical simulation. These solutions directly solve the differential equations of the SIR model, providing formulas for S(t), I(t), and R(t) as functions of time t. They make it possible to explicitly see how different parameters, like the transmission rate β, the recovery rate γ, and the basic reproduction number $R_{0}$, affect the disease dynamics.

#### 2.1.1. Estimation.

Estimation is crucial for inferring parameters in infectious disease models. This section covers two common methods: least squares estimation and maximum likelihood estimation.

##### 2.1.1.1. Least squares estimation.

A straightforward approach to estimating the parameters of compartmental models without making many assumptions is the least squares method. Taking the SIR model as an example, this method numerically integrates the differential equations across a range of β and γ values to generate theoretical curves for S(t), I(t), and R(t). Then these curves are compared with the observed data to calculate the sum of squared differences between the observed and theoretical values for each compartment. Parameter estimates are obtained by minimizing the objective function:

$${\text{argmin}}_{\beta ,\gamma }\sum_{t=1}^{T}[{\left(S_{\text{obs}}(t)-S_{\beta ,\gamma }(t)\right)}^{2}+{\left(I_{\text{obs}}(t)-I_{\beta ,\gamma }(t)\right)}^{2}+{\left(R_{\text{obs}}(t)-R_{\beta ,\gamma }(t)\right)}^{2}],$$

where t=1,…,T denote the total number of time points at which disease data were collected; $S_{\text{obs}}(t)$, $I_{\text{obs}}(t)$, and $R_{\text{obs}}(t)$ are the observed data for susceptible, infectious, and recovered individuals at time t; and $S_{\beta ,\gamma }(t)$, $I_{\beta ,\gamma }(t)$, and $R_{\beta ,\gamma }(t)$ are the theoretical values determined by the SIR model under specific parameters.

##### 2.1.1.2. Maximum likelihood estimation.

Unlike least squares estimation, which directly compares observed data with theoretical compartment values without assuming their probabilistic nature, maximum likelihood estimation introduces distributional assumptions about the observed data. For instance, we might assume that the observed count of infectious individuals at each time point follows a Poisson distribution, with the mean parameter defined by the SIR model’s theoretical number of infectious individuals for given β and γ values, i.e., $I_{\text{obs}}(t)∼\text{Poisson}(I_{\beta ,\gamma }(t))$. The likelihood function L(β,γ) is then expressed as

$$L(\beta ,\gamma )=\prod_{t=1}^{T}\frac{e^{-I_{\beta ,\gamma }(t)}{I_{\beta ,\gamma }(t)}^{I_{\text{obs}}(t)}}{I_{\text{obs}}(t)!}.$$

#### 2.1.2. Deterministic versus stochastic.

Continuing with the idea of incorporating probabilistic assumptions into infectious disease modeling, we turn to stochastic compartmental models. Traditional models like the basic SIR are deterministic, moving individuals between compartments at constant rates, resulting in smooth and predictable epidemic trajectories. This is effective for large populations where the law of large numbers smooths out random variations. However, deterministic models do not account for the inherent unpredictability of individual behavior, variability in disease transmission and recovery, or differing impacts of interventions. Additionally, precise tracking of actual numbers within compartments is rarely possible, highlighting the need for models that accommodate observational errors and uncertainty. In response, stochastic compartmental models emerged, incorporating randomness in disease transmission and recovery (Bartlett 1956, Matis & Wehrly 1979). This advancement provides a refined lens for viewing disease dynamics, especially for smaller populations or early outbreak phases. By embracing the probabilistic nature of infection and recovery events, stochastic models offer a spectrum of potential epidemic paths, quantify uncertainty, and reveal critical threshold phenomena that deterministic models cannot capture. Consequently, stochastic frameworks are indispensable for realistically simulating disease propagation, evaluating public health strategies, and enhancing decision-making under uncertainty. Focusing on the SIR model to illustrate a stochastic approach, we define the transition probabilities within a brief time interval dt as follows:

- Infection probability: The chance that a susceptible individual becomes infected in the interval dt is given by
$$P(\text{one}\;S\;\text{becomes}\;I\;\text{in d}t)=\beta \frac{I(t)}{N}S(t)dt.$$
- Recovery probability: The chance that an infected individual recovers in the interval dt is
P(oneIbecomesRin dt)=γI(t)dt.

Then the observed disease data over time can be viewed as outcomes of stochastic processes, and distributional assumptions can be made, such as the Poisson distribution for the number of new infections or recoveries. The model parameters then can be estimated using maximum likelihood or Bayesian approaches. For instance, assuming a Poisson distribution for the number of new infections and recoveries, the likelihood function for the stochastic SIR model, given a set of observed changes at discrete time points t=1,2,…,T, is

$$L(\beta ,\gamma )=\prod_{t=1}^{T}\left\{\frac{{\left(\beta \frac{I(t)}{N}S(t)dt\right)}^{\Delta I(t)}}{\Delta I(t)!}e^{-\beta \frac{I(t)}{N}S(t)dt}\right\}\cdot \left\{\frac{{\left(\gamma I(t)dt\right)}^{\Delta R(t)}}{\Delta R(t)!}e^{-\gamma I(t)dt}\right\}.$$

Here, ΔS(t), ΔI(t), and ΔR(t) denote changes in the number of susceptible, infected, and recovered individuals at time t, respectively. For details on stochastic compartmental models, including simulation and estimation approaches, readers are directed to Ganyani et al. (2021).

#### 2.1.3. Other extensions.

In recent decades, traditional compartmental models have evolved to capture the complex nature of disease transmission. These enhancements address diverse complexities such as heterogeneous susceptibilities, geographical spread, and the inherent randomness of transmission. Below, we explore these advanced extensions, highlighting their importance in refining our understanding and prediction of infectious disease dynamics.

##### 2.1.3.1. Age-structured models.

Age-structured models partition the population into distinct age groups, acknowledging that disease transmission and severity vary with age. These models are crucial for diseases like influenza or COVID-19, where age affects infection risk and outcomes. They are vital for designing targeted interventions, such as vaccination campaigns, and for evaluating the impact of nonpharmaceutical measures across different age groups (Truscott et al. 2009, Leung et al. 2021, Richard et al. 2021, de Miguel-Arribas et al. 2022, Iyaniwura et al. 2022).

For example, in an age-structured SIR model, the population is divided into distinct age groups to account for variations in contact rates, susceptibility, and recovery rates among different age demographics. For each age group a, let $S_{a}(t)$ denote the number of susceptible individuals, $I_{a}(t)$ the number of infectious individuals, and $R_{a}(t)$ the number of recovered individuals at time t. The dynamics of the disease transmission and recovery process in this framework can be described by the following set of differential equations:

$$\frac{dS_{a}(t)}{dt}=-\sum_{b=1}^{n}\beta_{ab}\frac{I_{b}(t)}{N_{b}}S_{a}(t),\;\frac{dI_{a}(t)}{dt}=\sum_{b=1}^{n}\beta_{ab}\frac{I_{b}(t)}{N_{b}}S_{a}(t)-\gamma_{a}I_{a}(t),\;\frac{dR_{a}(t)}{dt}=\gamma_{a}I_{a}(t),$$

where $\beta_{ab}$ represents the contact rate that results in transmission from individuals in age group b to individuals in group a, encapsulating the interaction patterns and efficiency of disease spread through contacts between different age groups; $\gamma_{a}$ is the recovery rate for individuals in group a, which reflects differences across age groups in immune response, access to medical care, or other factors that influence how quickly individuals recover from an infection; and $N_{b}$ is the total population in group b. This model accounts for varying interaction patterns between age groups, enabling detailed analysis of disease spread and the effectiveness of targeted interventions across demographics.

##### 2.1.3.2. Spatial models.

Spatial compartmental models extend traditional compartmental approaches by incorporating geographic locations into disease spread dynamics. This allows for studying transmission heterogeneity and simulating disease propagation across different regions. These models range from discrete approaches, where populations are divided into patches, to continuous models using geographic information systems (GIS) (Källén et al. 1985, Arino et al. 2007, Finger et al. 2016, Brauer et al. 2019, Aguilar et al. 2023, Maliyoni et al. 2023). Disease transmission within and between these locations is influenced by various factors, including population movement, local interactions, and spatial heterogeneity in disease parameters.

Taking a discrete spatial SIR model as an example, similar to traditional SIR models, each spatial patch has its own compartments for susceptible, infectious, and recovered individuals. This structure allows for modeling the local dynamics of disease transmission within each patch. Individuals can move between patches, simulating travel, migration, or daily commuting patterns. This movement is crucial for modeling the spread of disease between different geographic areas and can be represented by movement rates or probabilities that define how often individuals from one compartment in a patch move to another patch. The interaction between patches is modeled through coupling terms that represent the transmission of the disease due to the movement of people between patches. These terms can vary depending on the mobility patterns and the contact rates between individuals from different patches. Spatial compartmental models can incorporate variations in transmission rates, recovery rates, and other disease parameters across different patches. This heterogeneity can reflect differences in population density, local health policies, environmental conditions, and social behavior that affect disease spread.

Consider a spatial SIR model for two patches. The model is described by a set of differential equations that account for disease transmission within each patch, recovery, and the movement of individuals between patches. The equations for patch 1 are

$$\frac{dS_{1}(t)}{dt}=-\beta_{1}\frac{S_{1}(t)I_{1}(t)}{N_{1}}-m_{12}S_{1}(t)+m_{21}S_{2}(t),\;\frac{dI_{1}(t)}{dt}=\beta_{1}\frac{S_{1}(t)I_{1}(t)}{N_{1}}-\gamma_{1}I_{1}(t)-m_{12}I_{1}(t)+m_{21}I_{2}(t),\;\frac{dR_{1}(t)}{dt}=\gamma_{1}I_{1}(t)-m_{12}R_{1}(t)+m_{21}R_{2}(t),$$

and for patch 2,

$$\frac{dS_{2}(t)}{dt}=-\beta_{2}\frac{S_{2}(t)I_{2}(t)}{N_{2}}-m_{21}S_{2}(t)+m_{12}S_{1}(t),\;\frac{dI_{2}(t)}{dt}=\beta_{2}\frac{S_{2}(t)I_{2}(t)}{N_{2}}-\gamma_{2}I_{2}(t)-m_{21}I_{2}(t)+m_{12}I_{1}(t),\;\frac{dR_{2}(t)}{dt}=\gamma_{2}I_{2}(t)-m_{21}R_{2}(t)+m_{12}R_{1}(t),$$

where $\beta_{i}$, $\gamma_{i}$, and $N_{i}$ are the transmission rate, recovery rate, and total population in patch i, respectively. $m_{ij}$ represents the rate of movement from patch i to patch j. The coupling terms [$m_{21}S_{2}(t)-m_{12}S(t)$ for susceptibles in patch 1, and similarly for other compartments and patches] model the exchange of individuals between patches, thus incorporating the effect of human mobility on disease spread across the region.

A GIS-based SIR model takes a similar idea but integrates GIS data to continuously model locations. For example, a differential equation for susceptible individuals in a GIS-based SIR model can be written as

$$\frac{\partial S(x,y,t)}{\partial t}=-\beta (x,y)\frac{I(x,y,t)S(x,y,t)}{N(x,y)}+D_{S}\nabla^{2}S(x,y,t),$$

where S(x,y,t) and I(x,y,t) are the densities of susceptible, infectious, and recovered individuals at location (x,y) and time t, where x and y denote spatial coordinates. β(x,y) is now a function of location, reflecting how transmission may vary across different geographical areas due to local factors, e.g., population density, and public health interventions. $D_{S}$ is a diffusion coefficient for susceptible, representing the movement or spatial spread of individuals across the landscape. Higher values indicate greater mobility. $\nabla^{2}$ denotes the Laplace operator with respect to (x,y), which is used to model spatial diffusion (movement or spread) of each compartment across the geographic area. The differential equations for the other compartments can be constructed similarly.

Such spatial compartmental models can be further integrated with environmental, local demographic, health behavior, and mobility data to study how spatial heterogeneity is explained by environmental factors, regional characteristics, vaccination rates, and human mobility patterns within and between regions. These models are crucial tools for the evaluation of intervention impacts—like travel restrictions, localized lockdowns, and vaccination campaigns—allowing for the customization of public health strategies to specific regions or communities. By incorporating geographic differences, spatial compartmental models highlight the underlying factors that contribute to disparities in disease spread and outbreak severity across different areas. This makes them invaluable for informing and optimizing public health interventions and policies at both the local and regional levels.

##### 2.1.3.3. Network-based models.

Network-based compartmental models depict individuals as nodes and their interactions as edges in a network, facilitating detailed analysis of disease spread through social or contact networks (Miller et al. 2012, Ameri & Cooper 2019, Cheng et al. 2023, Das et al. 2023). To reflect the actual network contacts in a network-based SIR model, the term that represents the infection transmission rate needs to be adapted to account for the structure and the contacts within the network. In a homogeneous mixing model, the term βS(t)I(t)∕N assumes that each individual is equally likely to interact with any other individual in the population. However, in a network model, interactions are limited to those defined by the network’s edges.

As an example to think of this model, assume the population is composed of K distinct subpopulations, where each subpopulation k constitutes a proportion $w_{k}$ of the overall population, where k=1,…,K. Modeling the interactions between subpopulations using a network where edges define interactions between these subpopulations, we need to adjust the traditional SIR model to account for the network structure and the heterogeneity of contacts between different subpopulations.

Then the term βS(t)I(t)∕N in the fundamental SIR model, which assumes homogeneous mixing, needs to be adjusted to $\sum_{k=1}^{K}\beta_{kl}S_{k}(t)I_{l}(t)∕(Nw_{l})$ for each subpopulation k, where the sum is over all l subpopulations that have interactions (edges) with subpopulation k, and where $w_{l}$ represents the portion of individuals in subpopulation l relative to the total population size. This formula considers the interactions between different subpopulations and adjusts the infection rate according to the network’s structure. The adjusted term represents the rate at which individuals in subpopulation k are infected by individuals from subpopulation l, taking into account the size of subpopulation l and the number of infectious individuals within it. The division by $\left(Nw_{l}\right)$ is used to normalize the contact rate, assuming that the probability of contact with an infectious individual is proportional to the fraction of the subpopulation that is infectious. The summation over l accounts for the contributions of all infectious individuals from all subpopulations that have direct interactions with subpopulation k. It captures the network structure where not all subpopulations interact with each other with the same intensity.

The adjustment of the βS(t)I(t)∕N term to include network structure allows the model to capture heterogeneity in contact rates, $\beta_{kl}$, between different subpopulations and variability in disease spread dynamics due to the network of interactions between subpopulations, which can influence the effectiveness of public health interventions like vaccination or quarantine measures targeting specific subpopulations or connections. In practice, these network-based compartmental models can range from simple random networks to complex structures that closely resemble real-world social networks, incorporating community structures and varying degrees of connectivity. These models are critical for assessing the impact of network properties on disease transmission and for designing interventions like targeted vaccination or contact tracing.

##### 2.1.3.4. Multi-strain and vector-borne models.

For diseases involving multiple pathogens or transmitted by vectors, compartmental models have been extended to capture these additional layers of complexity. Multi-strain models consider the dynamics between multiple pathogens, including competition, coinfection, and cross-immunity effects (Minayev & Ferguson 2009, Levy et al. 2018, Khyar & Allali 2020, Massard et al. 2022), while vector-borne models include additional compartments for vectors, modeling the transmission cycle between hosts and vectors, which is essential for diseases like malaria or dengue (Wei et al. 2008, Lee et al. 2016, Cator et al. 2020).

For example, multi-strain SIR models are an extension of the basic SIR models designed to handle diseases that can exist in multiple strains within a population. This is particularly relevant for pathogens like influenza or COVID-19, where different strains may circulate simultaneously. The fundamental idea is to track the dynamics of each strain separately, considering cross-immunity and the potential for coinfection. The compartments in a multi-strain SIR model can include:

- S(t): Susceptible to all strains at time t
- $I_{i}(t)$: Infected with strain i at time t
- $R_{i}(t)$: Recovered from strain i, possibly with immunity to that strain at time t

The dynamics of the model for each strain i can be described by the transmission rate $\beta_{i}$ and recovery rate $\gamma_{i}$, adjusting the basic SIR equations to account for the presence of multiple strains.

Vector-borne SIR models are adapted to study diseases transmitted by vectors, such as mosquitoes in the case of malaria or dengue fever. These models include both the host (typically human) and vector populations, with compartments for susceptible, infectious, and recovered individuals within each population. The key addition is the interaction between hosts and vectors, requiring modifications to the transmission terms. For a vector-borne disease, a SIR model might include:

- For the host population: $S_{h}(t)$, $I_{h}(t)$, $R_{h}(t)$
- For the vector population: $S_{v}(t)$, $I_{v}(t)$

The transmission dynamics involve two rates: $\beta_{hv}$ for the transmission from infectious vectors to susceptible hosts and $\beta_{vh}$ for the transmission from infectious hosts to susceptible vectors. The force of infection in the host population then includes the interaction between susceptible hosts and infectious vectors, and vice versa for the vector population. The modified terms can be represented as:

- $\beta_{hv}$: The rate at which infectious vectors infect susceptible hosts
- $\beta_{vh}$: The rate at which infectious hosts infect susceptible vectors

For host infection by vectors, $\beta_{hv}\frac{S_{h}(t)I_{v}(t)}{N_{v}}$, where $N_{v}$ is the total vector population. For vector infection by hosts, $\beta_{vh}\frac{S_{v}(t)I_{h}(t)}{N_{h}}$, where $N_{h}$ is the total host population. The resulting model captures the cycle of transmission between hosts and vectors, essential for understanding and controlling vector-borne diseases.

##### 2.1.3.5. State-space models.

The SIR model can be cast within a state-space framework to study infectious disease dynamics, addressing uncertainties and time-varying elements in disease transmission and progression (Hooker et al. 2011, Osthus et al. 2017, Pei et al. 2018, Zhou & Ji 2020). Here, the compartments represent the state of the epidemiological system. If the actual number of susceptible, infected, and recovered individuals cannot be directly observed, a state-space version of the SIR model would use available data (like the number of new daily cases and recoveries) to estimate the underlying state.

The state equation models the true state’s evolution over time in a compartmental framework. For example, representing the state at time t as a vector $x_{t}={\left[S_{t},I_{t},R_{t}\right]}^{T}$ for susceptible, infectious, and recovered individuals, respectively, the state equation in a state-space SIR model is given by $x_{t+1}=F_{t}(x_{t},u_{t})+w_{t}$, where $F_{t}$ describes the state transitions, $u_{t}$ represents external influences, and $w_{t}$ is the process noise, capturing the randomness in state transitions. The observation equation relates the observable data to the underlying state with the observations at time t represented as $y_{t}$, and it is formulated as $y_{t}=H_{t}(x_{t})+v_{t}$, where $H_{t}$ maps the true state to observed data and $v_{t}$ represents measurement noise. The process noise, $w_{t}$, which models the uncertainty in disease state evolution, can be assumed to follow a Gaussian distribution with mean zero and covariance $Q_{t}$, i.e., $w_{t}∼𝒩(0,Q_{t})$. The observation noise, $v_{t}$, accounts for measurement errors, often modeled as Gaussian with mean zero and covariance $R_{t}$, i.e., $v_{t}∼𝒩(0,R_{t})$. Methods such as the Kalman filter for linear state-space models or the particle filter for nonlinear models like the SIR can be used to estimate the unobserved state variables ($S_{t}$, $I_{t}$, $R_{t}$) from incomplete and noisy data.

##### 2.1.3.6. Time-varying models.

One limitation of traditional compartmental models is their assumption of constant transmission rates throughout an epidemic. However, real-world outbreaks are influenced by dynamic factors that can significantly alter disease transmission rates over time. This motivated the development of time-varying compartmental models by adapting to dynamic changes in disease transmission rates over time, offering a more nuanced understanding of outbreaks (Chen et al. 2020, Zelenkov & Reshettsov 2023). For instance, time-varying SIR models extend the classic models by incorporating changing parameters over time. The differential equations are as follows:

$$\frac{dS(t)}{dt}=-\beta (t)\frac{S(t)I(t)}{N},\;\frac{dI(t)}{dt}=\beta (t)\frac{S(t)I(t)}{N}-\gamma (t)I(t),\;\frac{dR(t)}{dt}=\gamma (t)I(t).$$

Here, β(t) and γ(t) represent the time-varying transmission rate and the recovery rate, respectively, which are considered to be constant in the traditional SIR models. These parameters reflect how disease transmission and removal vary over time, influenced by factors such as public health interventions, changes in social behavior, and time-varying policies on testing and quarantine. The time-varying parameters can be modeled using various functions, depending on the characteristics of the outbreak and the nature of the data. Options include piecewise constant functions for distinct epidemic phases or smooth functions for gradual changes in transmission dynamics. Accurate estimation is challenging, especially with sparse or noisy data. A variety of techniques are available depending on the model and data, including finite impulse response filters and functional data approximation. Furthermore, methods like particle filtering or ensemble Kalman filtering facilitate the continuous updating of parameter estimates as new data become available.

#### 2.1.4. Strengths and limitations of compartmental models.

One of the primary strengths of compartmental models is their ability to simplify and structure the analysis of infectious disease dynamics by abstracting complex disease transmission mechanisms into a few compartments and parameters, providing a versatile and powerful tool for studying disease transmission. The models also enable the adaptation of the basic model structure to include more compartments or parameters tailored to specific diseases or conditions. Another advantage of compartmental models is their interpretation, including the interpretation of the contact rate and recovery rate, as well as the clear connection and interpretations of the basic reproductive number $R_{0}$, a key threshold parameter for understanding the disease and infectious pathogen. However, these models come with limitations. A fundamental assumption underpinning traditional compartmental models is that of homogeneity—an assumption that frequently fails to reflect the reality of diverse populations. This encompasses the ideas of homogeneous mixing, where each individual is presumed to have an equal opportunity to interact with others, and uniform susceptibility or infectiousness across the population. Moreover, while adding compartments to the model can enhance its ability to depict transmission dynamics more accurately, the increased complexity also results in a surge of parameters, which poses significant challenges to precise estimation.

### 2.2. Time-Since-Infection Models

Different from compartmental models, time-since-infection (TSI) models focus on the generation mechanism of infectious individuals, rather than the dynamics flow among multiple compartments of the population. They assume that all individuals newly infected at time t are infected by existing infectious individuals who could have been infected at any time before t, as shown in Figure 2. The concept of TSI models has its origins in the early twentieth century, specifically through the pioneering work of Ross and Hudson in the 1910s (Ross 1916; Ross & Hudson 1917a,b). Their mathematical basis was further elaborated in the landmark paper by Kermack & McKendrick (1927). The formalization and broader recognition of TSI models came with the contributions of Wallinga & Teunis (2004) and Fraser (2007). The models gained additional traction within the statistics community after Cori’s (2013) influential work, particularly with the development of the R package EpiEstim (Cori 2021), which facilitated their application in real-time estimation of disease transmission rates over the following decade.

During the COVID-19 pandemic, TSI models were widely applied to analyze the outbreak (Pan et al. 2020, Nouvellet et al. 2021, Amman et al. 2022, Nash et al. 2022, Ge et al. 2023). Moreover, recent advancements have been built on the foundation of the original TSI models. Researchers have introduced several refinements aimed at relaxing model assumptions or addressing challenges posed by real-world data. These enhancements range from integrating TSI models with regression techniques and tackling issues related to reporting delays and underreporting to improving the estimation of serial interval distributions and facilitating the concurrent analysis of data from multiple locations (Quick et al. 2021, Salas 2021, Wilder et al. 2021, Shi et al. 2022, Ge et al. 2023).

The simplification of the TSI model is made feasible by introducing biological information about infectious individuals. Specifically, it assumes that the infectiousness of an infected individual depends on the time since infection. Let β(t,s) denote the effective contact rate at time t between susceptible individuals and an infected individual who was infected s time ago, representing the number of secondary cases that an infected individual can generate at time t. At the population level, let $I_{t}$ denote the total number of new cases infected at time t, and then $I_{t}$ can be written as a function of the effective contact rate at time t and the number of all existing cases that were infected before time t, i.e., $E(I_{t})=E\int_{0}^{t}\beta (t,s)I_{t-s}ds$, where t≥0, and β(t,s)=0 for s∈(t,+∞).

In TSI models, the effective reproduction number is defined using the so-called instantaneous reproduction number, which measures the average number of susceptible individuals someone infected at time t could expect to infect if conditions remain unchanged. Mathematically, it is written as $R_{t}=\int_{0}^{\infty }\beta (t,s)ds$. Here, β(t,s) varies as the time since infection s changes, which typically reflects the change of pathogen shedding since infection. If we assume that the change of β(t,s) as a function of time since infection s is independent of calendar time t, then the β(t,s) can be decomposed as $\beta (t,s)=R_{t}\omega_{s}$. Here $\omega_{s}$ is a measure of infectiousness at the time since infection s, with $\int_{0}^{\infty }\omega (s)ds=1$, and $\omega_{s}=0$ for s=0 and s≥η, with η being the time to recovery since infection. It can be approximated by the density function of the distribution of generation time, which is defined as the time from the infection of a primary case to the infection of the secondary cases it generates, or the serial interval distribution, which represents the time between symptom onset of the infector and the infected.

For example, the shape of $\omega_{s}$ in Figure 3 assumes that the infectiousness of an infected individual increases during the first 3 days since infection as viral loads increase soon after infection and then gradually decrease as the individual proceeds to recovery about 15 days after infection. Such temporal patterns of infectiousness are related to viral shedding and have been reported in many infectious disease patients, including those with COVID-19 (He et al. 2020). Based on these assumptions, we have $E(I_{t})=R_{t}E\int_{0}^{t}\omega (t,s)I_{t-s}ds$. In practice, we can discretize $\left\{R_{t},t\geq 0\right\}$ and $\left\{\omega_{s},s\in (0,t]\right\}$ to be time series of equally spaced time points, where the time unit could be day, week, or month, such that

4. $$E(I_{t}|I_{0},\ldots ,I_{t-1})=R_{t}\Lambda_{t},$$

where $\Lambda_{t}=\sum_{s=1}^{t}I_{t-s}\omega_{s}$. To estimate $R_{t}$, it is natural to assume a distribution assumption based on Equation 4. For example, Cori et al. (2013) assumed a Poisson distribution, which is equivalent to model $\left\{I_{t},t\geq 0\right\}$ using a Poisson process. The model was used to study the epidemic of Ebola virus disease in West Africa (WHO Ebola Response Team 2014).

Compared with other methods, TSI models are notable for their practicality and flexibility. Being statistical in nature, they can integrate advanced statistical structures, making them ideal for modeling complex questions (Quick et al. 2021, Nash et al. 2022, Shi et al. 2022). TSI models are empirical and can be applied with a reasonable understanding of data generation, even without detailed epidemiological knowledge (Jewell 2021). This is particularly useful for modeling outbreaks of novel pathogens. Importantly, TSI models only require incidence data to estimate $R_{t}$, making them easy to estimate and user-friendly, especially when other surveillance data are unavailable. This simplicity is advantageous during early pandemic stages when surveillance systems are still developing. However, a significant weakness is their reliance on incidence data, which limits their ability to model or predict disease-related hospitalizations—a crucial metric for evaluating a pandemic’s trajectory and for hospital planning. This dependence also makes TSI models vulnerable to fluctuations in the quality of reported infection data.

### 2.3. Agent-Based Models

ABMs simulate the actions and interactions of autonomous agents, such as individuals or groups, to assess their effects on the system as a whole. ABMs are well-suited for modeling infectious disease spread, as they represent individual behaviors, heterogeneity, and local interactions in disease transmission. They track the spread of infection based on movements and contacts among susceptible, infected, and recovered individuals (Ferguson et al. 2005, Epstein et al. 2008, Kerr et al. 2021). While sharing some commonalities with network-based compartmental models, ABMs offer greater flexibility by providing a more detailed representation of individual behaviors and interactions, thus capturing complex transmission dynamics and the impacts of various interventions. The core components of ABMs in infectious disease modeling include the following.

- Agents: In infectious disease modeling, agents represent individuals or groups with attributes like age, health status, location, mobility patterns, and social connections. Agents can change states (e.g., susceptible to infected to recovered) based on interactions and disease transmission rules.
- Interactions: Disease transmission occurs through agent interactions, influenced by factors like physical proximity and social networks. Interactions can be dynamically updated to reflect behavioral changes (e.g., social distancing).
- Environment: The environment in which agents interact can range from abstract spaces to detailed geographic representations, including places like homes, schools, workplaces, and public spaces, influencing the pattern of contact and disease spread.
- Mobility: Agents can move within the environment according to predefined patterns or in response to conditions, e.g., seeking healthcare or adhering to lockdowns. Mobility patterns significantly affect the spread and control of infectious diseases.
- Rules: The behavior of agents and the progression of the disease are governed by a set of rules. These can include the probability of disease transmission upon contact, the duration of infectiousness, and behaviors like vaccination or mask-wearing.
- Time: ABMs typically simulate changes over time, either in discrete steps (time-steps) or as continuous updates. Each iteration represents the agents acting and interacting, leading to the evolution of the system’s state.

A conceptual diagram of an ABM for a small community with homes, schools, and workplaces is shown in Figure 4, illustrating agent interactions through direct contact or the environment. More complex models can incorporate additional agents, environments, and interaction rules. Mathematically, an ABM can be described by the states and rules that govern the behavior of each agent. Agents can be defined by state variables $s_{i}$ and a set of functions $f_{i}$ that describe the rules of behavior, where i indexes over all agents in the model. For example, an agent’s state at time t+1, $s_{i}(t+1)$, can be a function of its current state $s_{i}(t)$, the states of other agents $s_{-i}(t)$, and possibly some stochastic elements ϵ(t), i.e., $s_{i}(t+1)=f_{i}(s_{i}(t),s_{-i}(t),\epsilon (t))$. The model then progresses iteratively, with each agent updating its state based on its rules.

While ABMs focus on rules and interactions rather than traditional equations, they heavily rely on mathematical and statistical methods for their design, implementation, and analysis. Initial model conditions, including agent numbers, attributes, and environmental states, are often based on statistical distributions to reflect real-world variability. Agents’ actions, like deciding to vaccinate, can be modeled probabilistically, while mathematical functions represent movement or diffusion, affecting spatial interactions. Statistical methods are crucial for analyzing ABM outputs, such as estimating key epidemiological parameters, determining pattern significance, comparing scenarios, and fitting models to real-world data. Compared with mathematical models, ABMs offer a robust framework for understanding infectious disease spread by directly representing individual behaviors and interactions. They flexibly model interventions like vaccination, social distancing, and lockdowns to examine their impact on disease dynamics. ABMs capture heterogeneity in susceptibility, infectiousness, and behavior, providing insights into disease spread and control. However, ABMs are computationally intensive, requiring significant resources to simulate large populations with detailed interactions. They also rely on extensive data, which may not always be available, and face challenges in calibration and validation. Despite these challenges, ABMs offer valuable insights into public health interventions and support informed decision-making in outbreak response by accounting for the complexities of real-world populations.

### 2.4. Other Models

Besides the models described above, many other models have been employed to model infectious diseases, including time-series analysis, ML, and AI.

Time-series analysis offers a robust statistical framework for modeling and forecasting infectious disease trends over time by examining data points collected in time order and providing insights into the underlying patterns and potential future movements of disease spread (Held et al. 2005, Viboud et al. 2006, Dugas et al. 2013, Maleki et al. 2020, Liu et al. 2022, Zhang & Yi 2023). Several time-series models are commonly utilized for infectious diseases analysis. Autoregressive (AR) models, for instance, model current or future values based on past values, establishing a direct correlation between preceding and succeeding data points. Moving average (MA) models take a slightly different approach by leveraging past forecast errors in making future predictions. This methodology helps smooth out short-term fluctuations, offering a clearer view of the underlying trend. Integrated models, on the other hand, focus on differencing the data to achieve stationarity, a state in which a series’s statistical properties do not depend on time. This process is crucial for handling nonstationary data that exhibit trends or seasonality.

AR integrated MA (ARIMA) models combine the principles of AR, MA, and integration to address more complex time-series data comprehensively. This amalgamation allows ARIMA models to handle a variety of data structures, making them highly versatile in infectious disease modeling. For data with a seasonal pattern, seasonal ARIMA models extend the ARIMA framework to specifically account for seasonality, enhancing the model’s predictive accuracy for seasonal diseases. Lastly, vector autoregression models are employed for multivariate time-series data, capturing the interdependencies between multiple series. These models are particularly useful when dealing with diseases that may be influenced by or influence multiple factors simultaneously, offering a more holistic view of infectious disease dynamics. A major challenge of time-series analysis of infectious diseases is the assumption of stationarity, as many disease time series are nonstationary, making the selection of appropriate models critical to accurately capture the underlying trends and patterns. This often necessitates advanced statistical techniques to ensure model validity. Additionally, the complex dynamics of disease spread, influenced by numerous interrelated factors, may not be fully captured by time-series models alone, necessitating a comprehensive approach that incorporates multiple methodologies and data sources.

In recent years, the development of ML and AI has significantly transformed infectious disease modeling by enabling data-driven approaches that handle vast amounts of data, learn complex patterns, and predict future disease spread (Aramaki et al. 2011, Zoabi et al. 2021, Almotairi et al. 2023, Ye et al. 2023). ML models like long short-term memory networks forecast outbreaks by incorporating historical infection rates, mobility data, climate variables, and social media trends (Shahid et al. 2020). Techniques such as graph neural networks analyze contact tracing data to understand disease spread and identify high-risk nodes within social networks (Ng et al. 2022). ML models also assess disease spread risk using demographic data, health records, and environmental factors (Assaf et al. 2020), while AI models, particularly convolutional neural networks, enhance disease diagnosis through medical imaging (Elaziz et al. 2020). AI tools facilitate pathogen genome sequencing to track virus evolution and mutations (Yagin et al. 2023), and natural language processing techniques monitor social media for early outbreak detection and analyze public sentiment toward health policies (Aramaki et al. 2011, Hussain et al. 2021). Despite their potential, ML and AI models face challenges such as data privacy concerns, the need for large datasets, algorithmic bias, and transparency issues. Integrating ML and AI into infectious disease modeling offers promising tools to combat global health threats, provided their limitations and ethical implications are carefully considered through collaboration between data scientists, epidemiologists, and public health professionals.

### 2.5. Reproduction Numbers

Reproduction numbers are vital in epidemiology for understanding the spread of infectious diseases. They quantify the contagiousness or transmissibility of a pathogen. While there are several types of reproduction numbers, the most commonly discussed are the basic reproduction number $\left(R_{0}\right)$ and the effective reproduction number $\left(R_{t}\right)$.

#### 2.5.1. Basic reproduction number.

$R_{0}$ is the average number of new infections caused by a single infectious individual in a fully susceptible population during their infectious period. It measures the potential for disease spread. If $R_{0}>1$, each infected person, on average, infects more than one other person, leading to the potential for an epidemic. If $R_{0}<1$, the disease is likely to die out in the long term. $R_{0}$ is often estimated using models that consider the contact rate, transmission probability per contact, and duration of infectiousness. A commonly used definition is $R_{0}=\beta ∕\gamma$ based on the compartmental models, where β is the transmission rate per contact and γ is the recovery rate.

#### 2.5.2. Effective reproduction number.

$R_{t}$ represents the average number of secondary cases generated by an infectious individual at time t, considering the population’s current immunity and public health interventions. Unlike $R_{0}$, $R_{t}$ adapts to reflect changes in susceptibility, contact patterns, and interventions. Like $R_{0}$, if $R_{t}>1$, the epidemic is growing; if $R_{t}<1$, the epidemic is shrinking. $R_{t}$ provides a real-time snapshot of the transmission dynamics and is crucial for guiding public health interventions during an epidemic.

There is not a single, universal mathematical definition for $R_{t}$ due to its dependence on dynamic factors, but it can be conceptually understood and estimated through several methods. One common approach to define $R_{t}$ mathematically involves extending the concept of $R_{0}$, to account for the proportion of the population that remains susceptible at time t, among other factors, such that

$$R_{t}=R_{0}\frac{S(t)}{N}.$$

This definition implies that $R_{t}$ is directly proportional to $R_{0}$ and the proportion of the population that is still susceptible. However, this simplification does not capture all dynamics, such as changes in contact rates or the effects of interventions like social distancing and vaccination, which can also dramatically affect $R_{t}$. More sophisticated models calculate $R_{t}$ by using more complex models and adjusting the transmission rate over time, reflecting how changes in behavior, policy, and immunity affect disease spread. Examples include the effective reproduction number (Wallinga & Teunis 2004), the instantaneous reproduction number (Fraser 2007), the case reproduction number (Fraser 2007), and the time-dependent basic reproduction number (Boatto et al. 2018, Chen et al. 2020, Song et al. 2020). While the concept of $R_{t}$ is straightforward, its mathematical definition and calculation are complex and depend on dynamic factors including immunity and public health measures.

## 3. PREDICTIVE MODELING

Real-time projection of disease transmission is essential for developing effective mitigation strategies. Various models, including single, ensemble, global, and local models, provide diverse tools for predicting and managing outbreaks. Each model has its strengths and challenges, highlighting the importance of careful selection and application to enhance prediction accuracy and reliability.

### 3.1. Single Versus Ensemble Models

Single models rely on specific methodologies, data inputs, and assumptions to predict epidemic trends. They range from straightforward compartmental models like SIR to intricate ML algorithms, offering deep insights within their analytical scope. However, their predictive capabilities are inherently constrained by their singular approach and the assumptions on disease transmission dynamics and population behaviors they make. Ensemble models, by contrast, leverage the strengths of multiple predictive models to mitigate their individual weaknesses (Oidtman et al. 2021). Through techniques like weighted averaging, voting, and stacking methods, ensemble models amalgamate various predictions into a cohesive forecast (Ray & Reich 2018). This not only enhances the accuracy and reliability of the projections but also presents a more comprehensive strategy for managing the uncertainties intrinsic to epidemic modeling. The ensemble approach is distinguished by its ability to produce refined and robust predictions by drawing from the collective insights and diverse methodologies of several predictive frameworks.

### 3.2. Accuracy, Uncertainty and Goodness of Fit

Maintaining model accuracy in predictive modeling is an ongoing process that requires constant validation and refinement. These are crucial to ensure the model remains accurate as new data emerge and epidemic dynamics evolve. The approach includes assessing prediction accuracy, quantifying uncertainty, and focusing on goodness-of-fit diagnostics to identify areas for improvement. Validation involves the following:

- Comparing predicted versus observed outcomes: The model is regularly updated with recent data, and predicted events are compared with observed events to evaluate performance.
- Cross-validation techniques: Statistical methods like *k*-fold cross-validation are used to assess the model’s predictive power and generalizability by dividing the data into subsets and validating the model with one subset while training on the others.
- Sensitivity analysis: This involves examining how changes in model parameters or assumptions affect outcomes to identify the factors with the most significant impact on predictions.

Understanding and articulating the uncertainties in model predictions are also vital for interpreting their reliability. Doing so involves

- Identifying sources of uncertainty: These include variability in data quality, data gaps, model assumptions, and inherent randomness in disease transmission.
- Accounting for model uncertainty: Prediction intervals must reflect not just the variability observed in the data but also the uncertainties in the model, including the choice of model type, simplifications and assumptions, interactions between factors, parameter estimation methods, and model specification.
- Incorporating dependencies: In diseases with spatial and temporal dependencies, constructing prediction intervals must account for how correlations between data points affect the range of possible outcomes.
- Incorporating dynamic data and changing conditions: As new data come in and as interventions and public behaviors change, prediction intervals need to be continuously updated to reflect these dynamics accurately.

Additionally, assessing goodness of fit is crucial for identifying specific areas where the model performs well and where it may need improvement. This involves

- Residual analysis and diagnostic plots: examining the differences between observed and predicted values to detect any systematic biases or patterns that may indicate model inadequacies, and utilizing diagnostic plots, such as residuals versus fitted values and influence plots, to visually inspect the fit of the model and identify any deviations from expected behavior
- Model refinements: based on diagnostic findings, making targeted refinements to the model by iteratively adjusting assumptions, incorporating additional variables, or exploring alternative modeling approaches to enhance overall accuracy and reliability

### 3.3. Global Versus Local Models

Global models provide valuable insights on a macro scale, aiding in understanding broader pandemic trends and supporting international coordination. However, their generalized approach may overlook regional specifics, potentially affecting prediction accuracy at the community level. In contrast, local-level models offer more detailed predictions by considering local healthcare infrastructure, demographics, and other relevant factors. While they enhance accuracy locally, these models require comprehensive regional data and may struggle with generalizability across different settings.

### 3.4. Addressing Spatial and Temporal Correlation

Integrating spatial and temporal correlations into models significantly enhances their predictive power by accounting for the uneven spread of disease across different areas and over time. This approach is crucial for designing precise public health interventions and identifying transmission hot spots, enabling proactive strategies. Spatial correlation highlights the impact of geographical proximity on disease spread, while temporal correlation examines the epidemic’s progression over time. Models addressing both correlations can more accurately forecast disease spread by analyzing how changes in one area might influence another over time.

### 3.5. Adaptability and Scalability

The adaptability of models to new data and their scalability across various contexts are crucial for real-world application, which involves adjusting for data quality differences, incorporating diverse local factors, and using multi-level modeling techniques. Scalability can be enhanced by implementing a modular design, utilizing distributed computing, automating data integration, applying regularization techniques, and employing scalable algorithms. These strategies ensure models can handle large datasets, integrate new data seamlessly, and remain relevant and accurate across different regions and scales, from local to global levels.

### 3.6. Health Equity Considerations

Infectious disease models must address health equity to prevent worsening existing disparities. This involves accounting for diverse disease impacts across populations and ensuring public health recommendations are equitable and inclusive. Key steps include implementing inclusive data collection, integrating social determinants of health, and making equity-focused policy recommendations. These measures ensure models promote fairness and meet the needs of all population groups effectively.

## 4. IMPLEMENTATION

The two main approaches in infectious disease modeling are compartmental models and ABMs. Each has its strengths and weaknesses, necessitating careful consideration of best practices and sensitivity analysis. This section discusses their implementation and highlights key considerations.

### 4.1. Compartmental Models

Compartmental models vary in complexity, influenced by the number of compartments and their interactions. Thoughtful design of these compartments is crucial, requiring consideration of the specific research question, available data, and existing knowledge about the disease. Equally critical is the selection of estimation methods, as different methods may rely on varied assumptions and yield divergent estimates. Beyond parameter estimation, sensitivity analysis plays a pivotal role. This process involves systematically varying model parameters to assess how changes influence model outcomes. Sensitivity analysis illuminates which parameters are most significant, guiding where to focus efforts for improved data collection and parameter estimation. Furthermore, model validation and calibration are indispensable for ensuring the model’s fidelity to real-world data. Validating models against historical outbreak data and calibrating them using current, real-world data ensure that the model remains relevant and accurate over time.

### 4.2. Agent-Based Models

Similarly, ABMs range from very straightforward to highly complicated. They are particularly adept at integrating detailed behavior modeling through empirically based depictions of individual behaviors and interactions, enhancing the accuracy of disease spread simulations. This level of detail allows for the examination of how specific interventions or changes in behavior could impact an epidemic’s trajectory. However, they face significant challenges related to computational complexity and the need for detailed data. Simulating intricate behaviors and interactions demands substantial computational power, particularly for simulations on a large scale. Thus, ensuring scalability is paramount. These models need to be carefully engineered for efficient large-population simulations, balancing intricacy with computational pragmatism. Furthermore, the inclusion of spatial dynamics and mobility patterns adds realism to the models, providing a deeper understanding of disease transmission in realistic settings. However, generating such realistic simulations requires extensive data on individual behaviors and social networks, which can be challenging to gather.

### 4.3. Integrating Compartmental and Agent-Based Models

The integration of compartmental models and ABMs represents a powerful approach to infectious disease modeling, leveraging the strengths of each to provide both broad epidemiological trends and detailed insights into the mechanisms of disease spread. Hybrid models that combine the abstract structure of compartmental models with the detailed simulation capabilities of ABMs offer a versatile tool for exploring disease dynamics. Multi-scale modeling strategies, where ABMs address local dynamics and compartmental models capture global trends, present a promising avenue for bridging microlevel behaviors with macrolevel patterns.

In summary, the choice between compartmental models and ABMs, and the integration of both, or any other models, should be informed by the specific objectives of the study, the data availability, and the computational resources at hand. By adhering to best practices in model development and implementation—ranging from rigorous parameter estimation and sensitivity analysis to careful consideration of scalability and computational demands—researchers can effectively harness these models to gain deeper insights into infectious disease dynamics and support informed public health decision-making.

## 5. HEALTH POLICY EVALUATION

In the context of infectious disease modeling, evaluating the impact of health policies and interventions is crucial for guiding public health decisions, particularly in managing outbreaks and pandemics. Here, we discuss a few models that can be specifically tailored to understand the dynamics of infectious diseases and the effectiveness of strategies aimed at controlling their spread.

### 5.1. Cost-Effectiveness Analysis

Cost-effectiveness analysis (CEA) is a very useful tool in the analysis of infectious disease interventions, particularly when resources are scarce and critical decisions need to be made about the allocation of vaccines, treatments, and other health measures. The incremental cost-effectiveness ratio (ICER), a key metric in CEA, is defined as

$$ICER=\frac{C_{\text{new}}-C_{\text{standard}}}{E_{\text{new}}-E_{\text{standard}}},$$

where $C_{\text{new}}$ and $C_{\text{standard}}$ represent the costs of the new intervention and the standard (or no intervention) respectively, while $E_{\text{new}}$ and $E_{\text{standard}}$ denote their effectiveness, often measured in quality-adjusted life years. This ratio provides the additional cost per additional unit of health benefit gained from a new intervention compared with the standard, aiding in the prioritization of health policies that maximize health gains per unit of cost (Paul 2010, Horton 2017, Yuan & Li 2022).

### 5.2. Cost–Benefit Analysis

Cost–benefit analysis (CBA) becomes essential in analyzing the overarching impacts of pandemic response measures, balancing their economic costs against the benefits. In CBA, both costs and benefits are quantified in monetary terms, enabling a direct economic evaluation of interventions (Mohle-Boetani et al. 1995, Chaix et al. 1999, Rowthorn & Maciejowski 2020). The net benefit of an intervention is given by

NetBenefit=∑(Benefits−Costs),

where the benefits include reduced healthcare expenditures and economic impacts from averted illness and mortality, while costs encompass direct healthcare expenses and economic losses from interventions like lockdowns. This analysis informs policy decisions by evaluating if the monetary benefits of pandemic responses outweigh their costs.

### 5.3. Microsimulation Models

Microsimulation models simulate disease spread by representing individual behaviors and interactions over time, incorporating variables like demographics, contact patterns, and health states (Van der Ploeg et al. 1998, Reif et al. 2021, Spooner et al. 2021). They assess intervention impacts, such as social distancing and vaccination, on infection rates, hospitalizations, and mortality, providing detailed policy insights at both individual and population levels. While similar to ABMs, microsimulation focuses on evaluating outcome distribution and long-term policy effects using individual transitions based on statistical probabilities. In contrast, ABMs emphasize emergent behaviors from the interactions of autonomous agents, capturing complex system dynamics. Policymakers use these models for evidence-based strategies, optimizing resource use to control disease spread. By employing microsimulation models, health policymakers can derive evidence-based strategies for managing infectious diseases. Through the quantitative analysis offered by CEA and CBA, alongside the granular insights from microsimulation models, it is possible to identify and implement the most effective and efficient health interventions, ultimately aiming to control disease spread while optimizing resource use.

## 6. DATA REQUIREMENTS AND ETHICAL CONSIDERATIONS

The data requirements for infectious disease modeling are extensive, including case reports, demographic information, mobility patterns, genetic sequences, and healthcare capacity. Quality data are essential for accurately estimating disease parameters, predicting disease spread, evaluating intervention strategies, and informing public health decisions. However, obtaining accurate and timely data poses challenges due to underreporting, delays, and discrepancies in data management across regions. The dynamic nature of infectious diseases, with mutations and changing transmission dynamics, further complicates data collection and modeling. Addressing these challenges requires national and international collaboration to establish systematic data collection channels. Standardizing data reporting formats and frequencies can mitigate delays and discrepancies, enhancing the timeliness and comparability of information. Advanced genomic surveillance networks are essential for tracking pathogen evolution, allowing for rapid adjustments in response strategies. The use of personal and sensitive data, such as cell phone mobility traces and genetic information, introduces significant ethical and privacy considerations. Protecting individual privacy and ensuring data confidentiality are paramount. Anonymization is foundational, but robust data handling protocols and explicit consent mechanisms are necessary when detailed data are indispensable. Ethical considerations also include ensuring that modeling outcomes do not unjustly harm or stigmatize specific groups, avoiding contributing to health disparities or reinforcing social inequalities.

For infectious disease modeling to be both effective and ethical, a multifaceted approach to data collection, use, and management is necessary. This involves building comprehensive data infrastructures, fostering collaborative networks among governments, research institutions, and international organizations; ensuring strict data protection measures and transparency; and advocating for equity by using modeling outcomes to highlight and address disparities in disease impact and healthcare access.

## 7. FUTURE DIRECTIONS AND EMERGING TRENDS

The future of infectious disease modeling is set to advance significantly with developments in technology, data analytics, and interdisciplinary collaboration. This holistic approach integrates diverse data sources and expertise to address the multifaceted impacts of outbreaks on health, economies, and societies. In this section, we discuss key areas likely to shape the future:

- Enhanced integration of real-time data using ML and AI models: Real-time data analytics and big data technologies offer unprecedented opportunities for infectious disease modeling. High-resolution, real-time data from sources such as social media, mobile phones, and satellite imagery allow models to become more dynamic and responsive to changing outbreak conditions. By leveraging these data streams, future models can improve the timeliness and accuracy of predictions, facilitating more effective response strategies. The integration of ML and AI into infectious disease modeling enhances the potential of these real-time data sources. They can analyze complex datasets, identify patterns, and predict outbreak trends with high accuracy. They could also help decipher vast amounts of unstructured data generated during outbreaks, further enhancing predictive capabilities and enabling the identification of novel intervention strategies. Combining real-time data analytics with ML and AI methods will provide more accurate and timely insights, ultimately improving public health interventions and outcomes.
- Advances in genomic epidemiology: Genomic epidemiology is transforming infectious disease modeling by providing insights into the genetic mechanisms underlying disease transmission and pathogen evolution. Integrating genomic data with epidemiological models will help track pathogen mutations and spread, enabling precise identification of transmission pathways and assessment of control measures.
- Interdisciplinary collaboration: The complexity of infectious disease dynamics necessitates a multidisciplinary approach, bringing together expertise from epidemiology, statistics, biology, public health policy, and social sciences. This interdisciplinary collaboration is essential for developing models that capture the multi-dimensional impacts of outbreaks, including direct health effects, economic disruptions, and societal changes.
- Modeling multi-dimensional impacts: Several existing methods exemplify the trend toward modeling the multi-dimensional impacts of outbreaks. For example, multi-criteria decision analysis frameworks incorporate health outcomes, economic costs, and social implications to guide policy decisions. ABMs and microsimulation models provide granular insights into how individual behaviors and social networks influence disease spread and intervention effectiveness. Additionally, integrated assessment models from environmental science are being adapted to evaluate the interplay between human activities, environmental changes, and disease dynamics.
- Ethical and equitable modeling practices: Future models must address biases in data collection and analysis, ensuring predictions and interventions do not exacerbate health disparities. This will require including diverse populations in research and tailoring public health strategies to the needs of the most vulnerable. The future of infectious disease modeling is characterized by technological innovation, interdisciplinary collaboration, and a commitment to ethical and equitable health outcomes. Embracing these trends will enhance our ability to prevent, control, and mitigate infectious diseases globally.

## Figures and Tables

**Figure 1:**
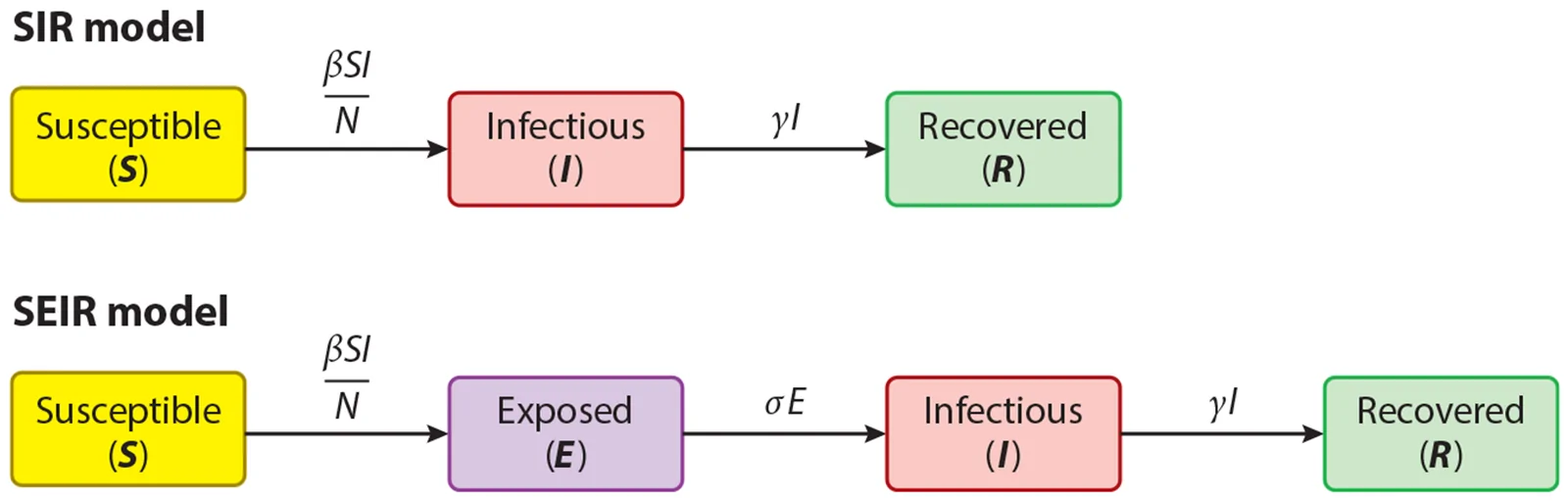
Illustration of two basic compartmental models: SIR (susceptible, infectious, recovered) and SEIR (susceptible, exposed, infectious, recovered) models. The SIR model delineates the progression of individuals through susceptible (S), infectious (I), and recovered (R) states, indicating direct transmission dynamics. The SEIR model introduces an additional exposed (E) state to account for the incubation period, capturing a delayed transition from exposure to infectiousness. Arrows represent the flow between compartments.

**Figure 2:**
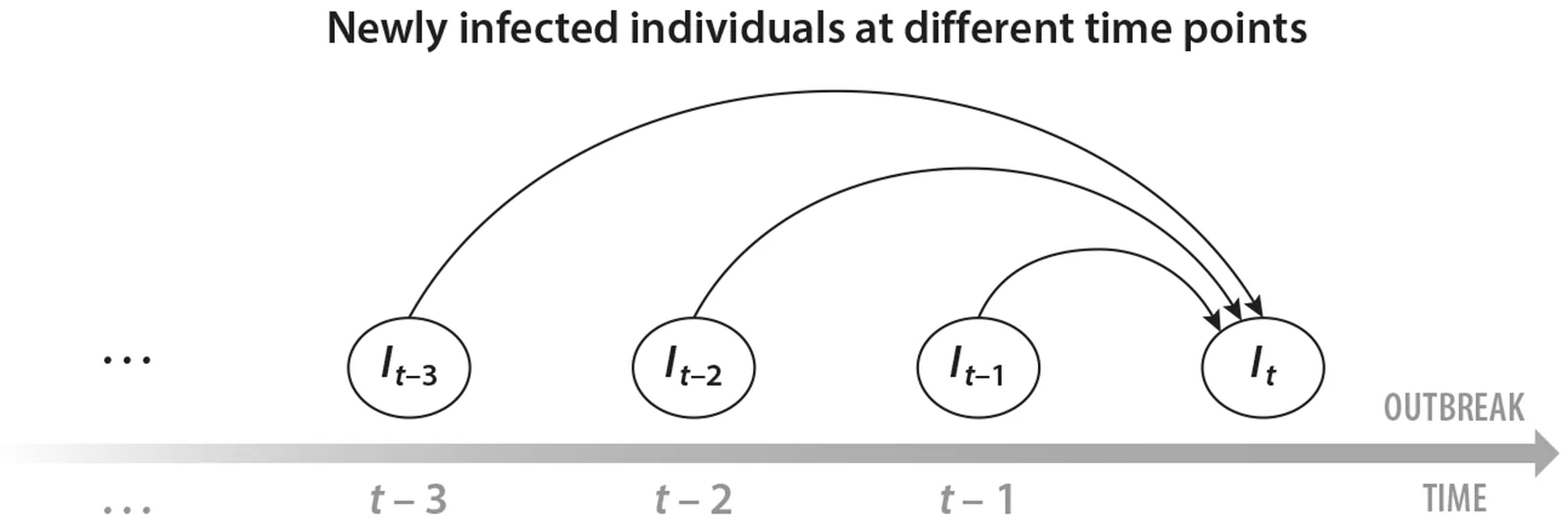
Illustration of the concept of the time-since-infection models. I(t) denotes the number of newly infected individuals at time t. The models focus on studying the generation mechanism of these individuals. It is assumed that all instances of I(t) are infected by existing disease carriers who could have been infected at any time before t, i.e., $I_{t-1},I_{t-2},\ldots ,I_{0}$. Moreover, the likelihood of being infected by an existing disease carrier is related to the infectiousness of the individual. This infectiousness, in turn, depends on the time since infection.

**Figure 3:**
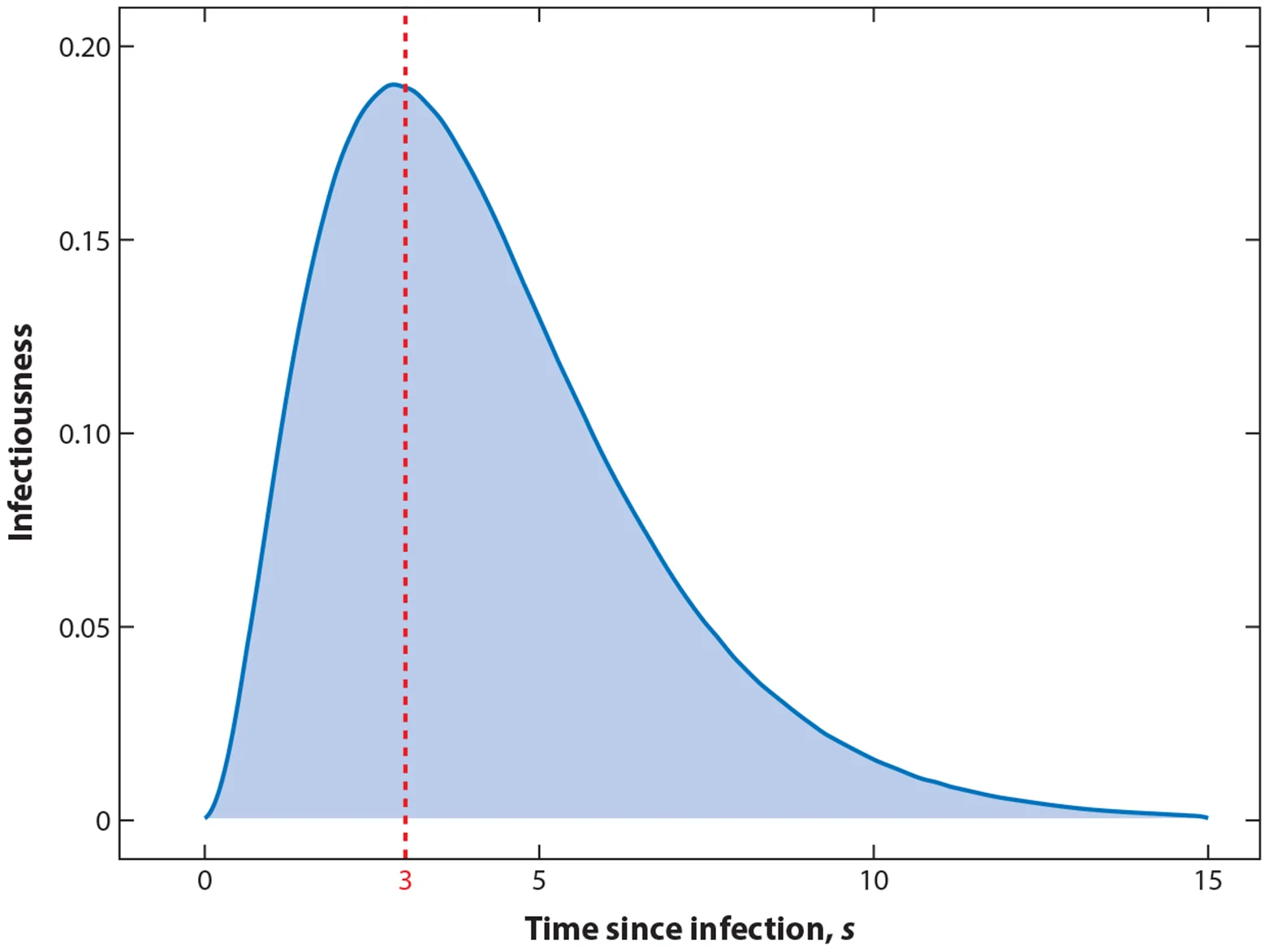
An example of measuring infectiousness at time s since infection, $\omega_{s}$, approximated using the density function of a gamma distribution with shape and rate parameters set at 3 and 0.7, respectively. Assuming time is measured in days, $\omega_{s}$ indicates that an infected individual’s infectiousness peaks during the first 3 days postinfection as viral loads surge shortly after infection. It then gradually declines as the individual progresses toward recovery, about 15 days after becoming infected.

**Figure 4:**
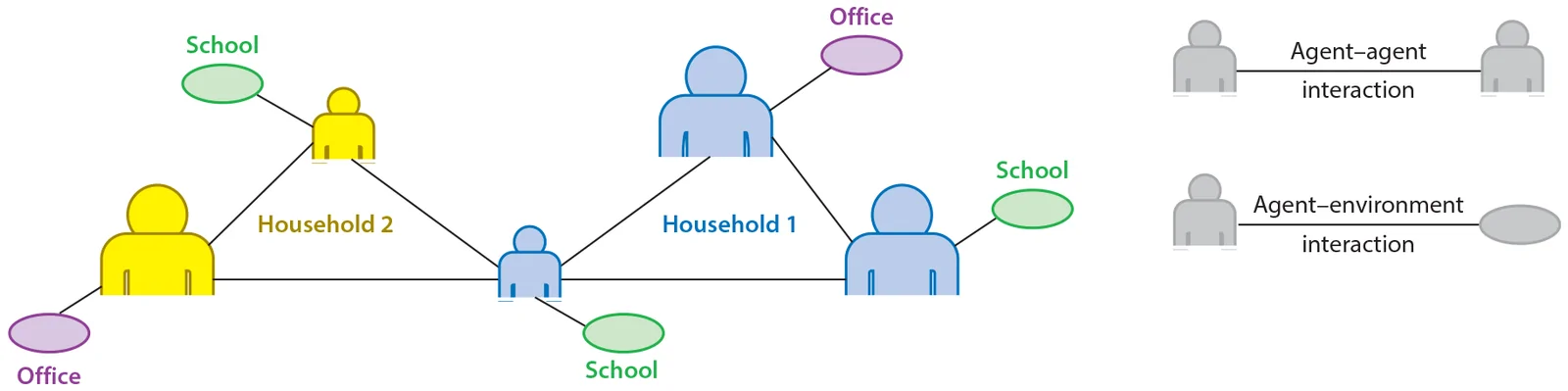
A conceptual diagram of an agent-based model for a hypothetical community includes two households, one school, and one office. Children interact at school, while adults interact at work.
